# rs1051931 Nonsynonymous Polymorphism of Platelet-Activating Factor Acetylhydrolase Gene *PLA2G7* Is Associated with Dysesthesia and Pain Severity After Surgery

**DOI:** 10.3390/ijms26093931

**Published:** 2025-04-22

**Authors:** Mayuko Hayashi, Seii Ohka, Daisuke Nishizawa, Rie Inoue, Masakazu Hayashida, Junko Hasegawa, Kyoko Nakayama, Yuko Ebata, Yuna Kang, Kaori Yoshida, Kyotaro Koshika, Ken-ichi Fukuda, Tatsuya Ichinohe, Kazutaka Ikeda

**Affiliations:** 1 Addictive Substance Project, Tokyo Metropolitan Institute of Medical Science, Setagaya-ku, Tokyo 156-8506, Japan; hayashi-my@igakuken.or.jp (M.H.); nishizawa-ds@ncnp.go.jp (D.N.); mhaya@juntendo.ac.jp (M.H.); hasegawa-jk@igakuken.or.jp (J.H.); nakayama-kk@igakuken.or.jp (K.N.); kang-yn@igakuken.or.jp (Y.K.); yoshidakaori@tdc.ac.jp (K.Y.); 2 Department of Dental Anesthesiology, Tokyo Dental College, Chiyoda-ku, Tokyo 101-0061, Japan; koshikakyotarou@tdc.ac.jp (K.K.); ichinohe@tdc.ac.jp (T.I.); 3 Department of Neuropsychopharmacology, National Institute of Mental Health, National Center of Neurology and Psychiatry, Kodaira, Tokyo 187-8551, Japan; 4 Department of Anesthesiology and Pain Medicine, Juntendo University Graduate School of Medicine, Bunkyo-ku, Tokyo 113-8421, Japan; 5 Department of Oral Health and Clinical Science, Tokyo Dental College, Chiyoda-ku, Tokyo 101-0061, Japan; kfukuda@tdc.ac.jp

**Keywords:** *PLA2G7*, SNP, PAF, PAF-AH, postoperative pain, dysesthesia

## Abstract

Platelet-activating factor (PAF) is a potent inflammatory mediator that activates the PAF receptor, which induces additional PAF production. Animal studies have shown that PAF induces inflammatory and neuropathic pain, including dysesthesia, a prodromal symptom of neuropathic pain. However, in humans, the association between PAF and pain remains unknown. Phospholipase A2 Group VII (PLA2G7) hydrolyzes PAF to eliminate PAF activity. The present study investigated the association between the *PLA2G7* rs1051931 nonsynonymous polymorphism (T/C, Val379Ala), which decreases the PAF-degrading activity of PLA2G7 in plasma, and postoperative pain-related phenotypes in humans. The study included 303 patients who underwent sagittal split ramus osteotomy at Tokyo Dental College and were assessed for dysesthesia and 332 patients who underwent laparoscopic gynecologic surgery at Juntendo University Hospital and were assessed for postoperative pain using the Numeric Rating Scale (NRS). *PLA2G7* rs1051931 was significantly associated with dysesthesia (*p* = 0.0491) and NRS scores (*p* = 0.0243). Carriers of the CC genotype of *PLA2G7* rs1051931 were more likely to have dysesthesia and higher NRS scores than carriers of the TT + TC genotypes. Carriers of the CC genotype of *PLA2G7* rs1051931 reportedly had lower PAF-degrading activity in plasma, thereby increasing the amount of PAF. The increase in PAF possibly leads to dysesthesia and postoperative pain in humans.

## 1. Introduction

Acute postoperative pain is classified into nociceptive pain, inflammatory pain, and pathological pain, including neuropathic pain [1]. Acute postoperative pain sometimes leads to chronic postsurgical pain [2], which is often caused by neuropathic pain (in an average of 30% of cases) [3]. This form of pain, including the neuropathic component, is usually more severe than nociceptive pain and often impairs quality of life [4].

Neuropathic pain is caused by damage or dysfunction of the peripheral or central nervous system and is highly individualized and intractable. Sensory disturbances, such as dysesthesia and hypoesthesia, often appear as prodromal symptoms of neuropathic pain. Sagittal split ramus osteotomy (SSRO) and laparoscopic surgery for benign gynecological diseases involve nerve compression or damage. In addition to acute postoperative pain, including nociceptive and inflammatory pain, nerve compression or damage causes sensory disturbances in SSRO [5,6] and neuropathic pain in laparoscopic gynecological surgery [7].

Few reports currently show an association between sensory disturbances and gene polymorphisms. For sensory disturbances after SSRO, dysesthesia was reportedly associated with a nonsynonymous genetic polymorphism of the *METTL4* gene, and hypoesthesia was associated with genetic polymorphisms in flanking regions of the *ARID1B* and *ZPLD1* genes [8]. For postoperative pain after SSRO, there are no reports of an association between postoperative pain (i.e., neuropathic pain) after SSRO and gene polymorphisms other than the association between the analgesic effect of fentanyl on postoperative pain and gene polymorphisms of *OPRM1* [9,10]. For postoperative pain after laparoscopic gynecological surgery, associations between postoperative pain after laparoscopic gynecological surgery and gene polymorphisms of the *SCN9A* and *SCN11A* genes, both related to neuropathic pain, have been reported [11,12,13,14]. The *METTL4* gene encodes methyltransferase-like protein 4. The *OPRM1* gene encodes the μ-opioid receptor. The *SCN9A* and *SCN11A* genes encode voltage-gated sodium channel proteins type 9 subunit α and type 11 subunit α, respectively. The *ARID1B* gene may affect cell cycle activation, and the function of the *ZPLD1* gene is unknown. The relationship between these genes and PAF has not been reported.

Platelet-activating factor (PAF) is a lipid mediator that exerts various biological effects through the PAF receptor (PAFR). Immune cells, such as macrophages and microglia, produce PAF [15]. With regard to signaling pathways that are involved in pain or inflammation, PAF/PAFR interaction increases intracellular calcium concentrations [16], leading to adenosine triphosphate (ATP) release and the activation of ATP receptors on sensory neurons to cause pain [17,18] and on macrophages or microglia to induce inflammation [19,20,21]. Phospholipase A2 on the cell membrane converts a biologically inactive precursor into lyso-PAF, which is then converted to PAF by acetyltransferase LPCAT1 or LPCAT2 [22]. PAF is subsequently degraded by PAF acetylhydrolase (PAF-AH/Lp-PLA2). PAF-AH is classified into three types: intracellular PAF-AH I and PAF-AH II and extracellular plasma-type PAF-AH. Phospholipase A2 group VII (PLA2G7), encoded by the *PLA2G7* gene, is a plasma-type PAF-AH that degrades PAF to biologically inactive products.

In mice, PAF that is produced by macrophages and microglia activates the PAFR on itself, robustly enhancing PAF production [15]. The increase in PAF production causes neuropathic pain through the PAFR on primary sensory neurons, leading to the PAF–pain loop [15]. The PAF/PAFR system contributes to acute nociceptive and inflammatory pain, chronic pain (including neuropathic pain) [23,24], inflammatory responses [25], mechanical allodynia, and hyperalgesia in animal models [17]. PAF also exacerbates nociceptive and inflammatory postoperative pain [26]. In humans, plasma PAF-AH activity decreased in patients with inflammatory conditions, such as active systemic lupus erythematosus, necrotizing enterocolitis [27], bronchial asthma [28], sepsis [29], and severe anaphylaxis [30], compared with healthy individuals, implying an increase in plasma PAF levels through a decrease in PAF-AH activity that may contribute to the pathogenesis of these inflammatory responses. For the association between PAF and pain, high plasma PAF levels correlated with the severity of menstrual pain in primary dysmenorrhea [31]. Notably, the activation of the PAFR amplifies pain in fibromyalgia, which has an element of neuropathic pain [32]. However, the direct association between plasma PAF-AH activity or plasma PAF concentrations and postoperative pain, including neuropathic pain, has not been reported in humans.

The *PLA2G7* gene is reportedly associated with cardiac disease and stroke [33], but there are no reports of an association with pain. The rs1051931 T/C single-nucleotide polymorphism (SNP) of the *PLA2G7* gene leads to an amino acid substitution (Val379Ala) of PLA2G7, and the PLA2G7 protein with Ala at 379 position was shown to result in a 2.7% decrease in PAF-AH activity in an aliquot of plasma in humans [34], although it is unclear whether the decrease in PAF-AH activity is attributable to a decrease in the PAF-AH enzymatic activity of PLA2G7 or a decrease in the mass of PLA2G7. Together with the facts that a decrease in plasma PAF-AH activity is related to inflammatory responses in humans [27,28,29], PAFR activation or high PAF levels are associated with menstrual pain in primary dysmenorrhea and fibromyalgia in humans [31,32], and an increase in PAF production exacerbates postoperative pain (including neuropathic pain) in mice [15,26], the decrease in PAF-AH activity in plasma caused by the *PLA2G7* rs1051931 polymorphism may contribute to acute postoperative pain, including neuropathic pain, in humans through higher plasma levels of PAF.

In the present study, we investigated the association between the rs1051931 polymorphism of the *PLA2G7* gene, which decreases PAF-AH activity in plasma in humans, and dysesthesia after SSRO and pain severity after laparoscopic gynecologic surgery to clarify the association between PAF-AH activity and acute postoperative pain, including neuropathic pain-related dysesthesia, in humans.

## 2. Results

### 2.1. PLA2G7 rs1051931 Is Associated with Dysesthesia in the TD Group

Associations between the rs1051931 SNP of the *PLA2G7* gene and the dysesthesia phenotype in 303 patients who were scheduled to undergo SSRO at Tokyo Dental College (TD group) were analyzed. The patients’ genotype distributions of the rs1051931 SNP of *PLA2G7* are shown in Table 1.

The *PLA2G7* rs1051931 genotype distribution did not deviate from the theoretical Hardy–Weinberg equilibrium (*χ*^2^ = 1.084, *p* = 0.298). Pearson’s *χ*^2^ test revealed a significant difference in the dysesthesia phenotype in the dominant model (between the TT + TC and CC genotypes), suggesting that *PLA2G7* rs1051931 is significantly associated with dysesthesia in our samples (*p* = 0.049; Table 2). No significant differences in dysesthesia were observed in the genotypic model (among the TT, TC, and CC genotypes) or recessive model (between the TT and TC + CC genotypes) of *PLA2G7* rs1051931 (*p* > 0.05; Table 2). In the dominant model of *PLA2G7* rs1051931, the rate of the CC genotype was higher in patients with dysesthesia compared with patients without dysesthesia (rate of CC genotype: with dysesthesia, 86.6%; without dysesthesia, 77.9%; Table 2).

Thus, the rate of CC genotype was higher in patients with dysesthesia. This result suggests that patients with the CC genotype of *PLA2G7* rs1051931 are more likely to suffer dysesthesia. To ascertain linearity, the Cochran–Armitage trend test was applied. The rate of the TT, TC, and CC genotypes in patients with dysesthesia were 0%, 13.4%, and 86.6%, respectively. The rate of the TT, TC, and CC genotypes in patients without dysesthesia were 0.7%, 21.4%, and 77.9%, respectively (Table 2). The patients’ genotypic groups showed significant linearity according to the Cochran–Armitage trend test (*p* = 0.049), indicating a tendency toward dysesthesia as the number of C alleles in the genotypes increased.

### 2.2. PLA2G7 rs1051931 Is Associated with Numeric Rating Scale Pain Scores in the JUH Group

Associations between *PLA2G7* rs1051931 and Numeric Rating Scale (NRS) pain scores in 332 patients who underwent laparoscopic surgery for benign gynecological disease were analyzed. The patients’ genotype distributions of *PLA2G7* rs1051931 are shown in Table 1. *PLA2G7* rs1051931 did not deviate from the theoretical Hardy–Weinberg equilibrium (*χ*^2^ = 0.616, *p* = 0.432). In the genotypic model (among the TT, TC, and CC genotypes), the Kruskal–Wallis test revealed that *PLA2G7* rs1051931 showed a trend toward significance in the association with NRS pain scores (genotypic model, *p* = 0.076, Table 3).

In the dominant model (between the TT + TC and CC genotypes), the Mann–Whitney *U* test showed a significant association between *PLA2G7* rs1051931 and NRS pain scores (dominant model, *p* = 0.025; Table 3), suggesting that *PLA2G7* rs1051931 is significantly associated with pain in the dominant model. In the recessive model (between the TT and TC + CC genotypes) of *PLA2G7* rs1051931, NRS pain scores did not show a significant association (*p* > 0.05; Table 3).

**Figure 1:**
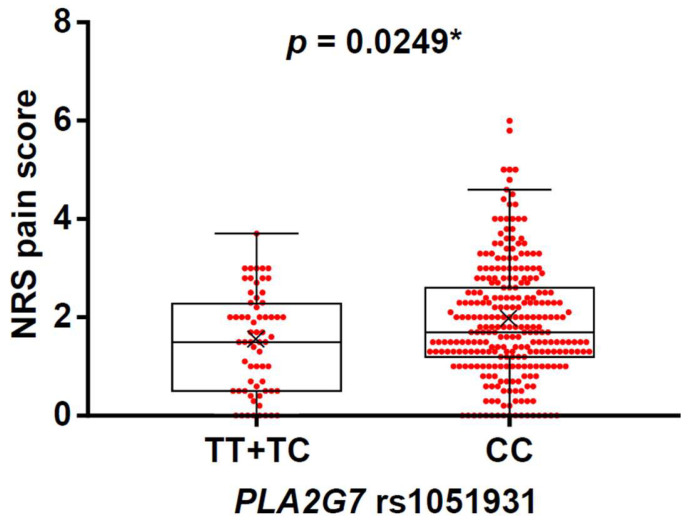
Associations between genotypes of *PLA2G7* rs1051931 and NRS pain scores in the JUH group. The data are expressed as box and whisker plots. The upper and lower ends of the boxes represent the 75th and 25th percentiles, respectively. Whiskers represent the highest and lowest values. Medians are depicted by solid horizontal lines in the boxes. Mean values are depicted by crosses. Each NRS value is depicted by a red dot. The samples were divided into two groups in the dominant model (TT + TC and CC). TT + TC, *n* = 64; CC, *n* = 268; * *p* < 0.05.

For median NRS pain scores in genotypes of *PLA2G7* rs1051931, median NRS pain scores of the TT, TC, and CC genotypes were 1.3, 1.5, and 1.7, respectively. To ascertain linearity, the Jonckheere–Terpstra trend test was applied. The genotypic groups showed significant linearity in median NRS pain scores for patients’ genotypic groups (*p* = 0.024, Jonckheere–Terpstra trend test; Table 3). This result suggests that pain severity becomes higher as the number of C alleles in the genotypes increases. In the dominant model of the rs1051931 SNP, median NRS pain scores were significantly higher in the CC genotype compared with the TT + TC genotype (median NRS pain score: TT + TC, 1.5; CC, 1.7; Figure 1). This result suggests that patients with the CC genotype are more likely to have higher pain severity.

## 3. Discussion

The present study examined the impact of the rs1051931 SNP of the *PLA2G7* gene on postoperative pain-related phenotypes in patients who underwent SSRO and laparoscopic gynecological surgery. In patients who underwent SSRO, the presence or absence of dysesthesia was evaluated. In patients who underwent laparoscopic gynecological surgery, postoperative pain within 24 h was assessed. Carriers of the CC genotype of *PLA2G7* rs1051931 were more prone to the development and exacerbation of dysesthesia and postoperative pain than those with TT + TC genotypes. These results suggest that the CC genotype of *PLA2G7* rs1051931 is associated with postoperative pain, including neuropathic pain.

In animal models, PAF was shown to induce new PAF production in a feedback loop to exacerbate neuropathic pain [15]. The administration of a small amount of PAF into the spinal cord in mice caused allodynia, which is related to neuropathic pain, and hyperalgesia [17]. Furthermore, the intrathecal spinal administration of a PAFR antagonist alleviated neuropathic pain [17]. PAF also exacerbates nociceptive and inflammatory postoperative pain [26]. These findings align with the present results in humans, in which CC carriers of *PLA2G7* rs1051931, which is related to a decrease in PAF-AH activity in plasma, were associated with postoperative pain, including neuropathic pain. Thus, the increase in PAF through a decrease in PAF-AH activity may contribute to the development and exacerbation of a prodromal symptom of neuropathic pain, dysesthesia, and postoperative pain in humans.

PAF-AH degrades PAF, which is involved in neuropathic pain [15]. The base substitution from T to C in the rs1051931 polymorphism of the *PLA2G7* gene causes an amino acid substitution from Val to Ala of PLA2G7, plasma type PAF-AH [35]. CC carriers of the *PLA2G7* rs1051931 polymorphism have reportedly exhibited a decrease in PAF-AH activity in an aliquot of plasma [34,36,37,38], with the exception of one study [39]. Qi et al. reported that mean PAF-AH activity in an aliquot of plasma was TT (Val/Val) > TC (Val/Ala) > CC (Ala/Ala) [37], and Hoffmann et al. reported TT > TC + CC [38]. However, we cannot discern the reason for the lower PAF-AH activity in an aliquot of plasma (i.e., whether it is attributable to a decrease in PAF-AH activity of *PLA2G7* or a decrease in *PLA2G7* mass) that depended on genotypes of *PLA2G7* rs1051931. Qi et al. indicated that the mean mass of PAF-AH in plasma was TT > TC > CC [37]. Similarly, the result of expression quantitative trait locus (eQTL) by a public GTEx portal database indicates that *PLA2G7* mRNA expression in AA, AG, and GG genotypes of *PLA2G7* rs1051931 (TT, TC, and CC genotypes according to the orientation of the *PLA2G7* gene) is of a reduced order in a linear regression model (normalized effect size (NES) = −0.25, *p* = 5.56 × 10^7^, Supplementary Figure S1) [40]. These findings indicate that the mass of plasma-type PAF-AH/PLA2G7 is TT > TC > CC, resulting in the amount of PAF in plasma being TT < TC < CC. Although we do not know whether the amino acid substitution causes a decrease in the PAF-AH enzymatic activity of PLA2G7, it is possible that PAF-AH activity in an aliquot of plasma decreases in the order of TT > TC > CC, and the concentration of PAF would be TT < TC < CC, which is consistent with the clinical findings in the present study, in which the development of dysesthesia and postoperative pain were TT < TC < CC.

Microglia and macrophages are involved in neuropathic pain [41,42] and are potential PAF-producing cells that are involved in the onset of neuropathic pain in mice [43]. In the public GTEx Portal database, eQTL results of the *PLA2G7* rs1051931 SNP showed that genotypes of *PLA2G7* rs1051931 affect *PLA2G7* mRNA expression levels in the basal ganglia [40]. Another public database, the Human Protein Atlas, showed that macrophages highly express *PLA2G7* in both the central nervous system and blood, and microglia express *PLA2G7* [44]. Although PAF and PLA2G7 do not penetrate the blood–brain barrier (BBB) [45], PAF is produced and PLA2G7 is expressed even inside the BBB [46], probably by microglia and macrophages. The increase in local concentrations of PAF inside the BBB that depends on genotypes of *PLA2G7* rs1051931 may stimulate microglia and macrophages in an autocrine and paracrine manner, which would lead to an inflammatory response and the exacerbation of pain in humans.

Public data extraction using the ZENBU genome browser revealed that the *PLA2G7* rs1051931 SNP is located around 300–500 bp upstream of the enhancer region in immune cells and the brain, including CD14 primary cells, the anterior caudate, and the dorsolateral prefrontal cortex (Supplementary Table S1) [47]. DNase-Seq results showed that the *PLA2G7* rs1051931 SNP is located around 300–500 bp upstream of the genome region with high signals on DNase-Seq on the chromosome of immune cells (score > 15: CD14 and CD15 blood primary cells and CD14 monocytes), and relatively high signals detected in many tissues, including in the brain (Supplementary Table S2) [47]. DNase-Seq signals indicated that the regions contain open chromatin and are thought to contribute to transcription. CD14 is expressed in both microglia and macrophages and plays an essential role in brain injury in these cells [48,49]. CD14-positive immune cells, including macrophages, highly express *PLA2G7* [50,51,52]. The regions on the chromosome downstream of the *PLA2G7* rs1051931 SNP would contribute to transcriptional activity of *PLA2G7* as an enhancer, mainly in CD14-positive immune cells including microglia/macrophages. Together with the fact that CD14-positive cells play a crucial role in the expression of pain-related molecules in human degenerated intervertebral discs [53], PLA2G7 expression in CD14-positive cells, including microglia/macrophages, would contribute to pain development, depending on genotypes of the *PLA2G7* rs1051931 SNP, through PAF-AH activity.

In the present study, the allele frequencies of the *PLA2G7* rs1051931 SNP in different regional populations were the following. In the TD group, the frequency of the T allele was 9% and the frequency of the C allele was 91%. In the JUH group, the T allele frequency was 10%, and the C allele frequency was 90%. According to the 1000 Genomes Project SNP database [35,47,54], the T allele frequency in East Asian populations is 9% and the C allele frequency is 91%. In South Asian populations, the T allele frequency is 14% and the C allele frequency is 86%. In American populations, the T allele frequency is 15% and the C allele frequency is 85%. In African populations, the T allele frequency is 28% and the C allele frequency is 72%. In European populations, the T allele frequency is 24% and the C allele frequency is 76%. Based on this information, the higher frequency of the C allele in Japanese and East Asian populations than in other populations suggests a potentially higher risk of developing postoperative pain, including neuropathic pain, in these populations in terms of the *PLA2G7* rs1051931 SNP.

The present study has a few limitations. First, analgesics that were administered after surgery varied among patients in the JUH group. The variable use of analgesics could affect NRS pain scores. Second, female patients predominated in both the TD and JUH groups (62% and 100%, respectively). The results in the present study may mainly reflect phenomena in females.

## 4. Materials and Methods

### 4.1. Ethics Statement

The study protocol was approved by the Institutional Review Board at Tokyo Dental College (approval no. 812-2, date of approval: 29 July 2024), the Juntendo University Faculty of Medicine (approval no. 2015053, date of approval: 18 September 2015), and the Tokyo Metropolitan Institute of Medical Science (approval no. 23–32 and 21–26(1), dates of approval: 29 March 2024 and 13 February 2024).

All subjects provided written consent after being informed about participating in the genetic studies.

### 4.2. Subjects

#### 4.2.1. Patients Who Underwent SSRO

The study population for the association study of *PLA2G7* gene polymorphisms and dysesthesia included 303 healthy patients (American Society of Anesthesiologists Physical Status I; age, 15–50 years; 116 males and 187 females) who were scheduled to undergo SSRO for mandibular prognathism at Tokyo Dental College, Suidobashi Hospital, as described previously (303 patients whose data on dysesthesia were available out of 361 patients in the TD group [8]). Briefly, patients with obvious nerve damage during surgery were excluded. For the 303 Japanese patients who underwent SSRO for mandibular prognathism, the recruitment of subjects, surgical protocol, and subsequent postoperative pain management were fundamentally the same as in a previous report [8]. Detailed demographic and clinical data of the subjects are provided in Supplementary Dataset S1.

#### 4.2.2. Patients Who Underwent Laparoscopic Surgery for Benign Gynecological Disease

The association between the *PLA2G7* rs1051931 polymorphism and NRS scores was examined in patients (American Society of Anesthesiologists Physical Status I~II; age, 20–70 years; 333 females) who were scheduled to undergo laparoscopic surgery for benign gynecological diseases at Juntendo University Hospital (JUH group [55]). Patients who were chronically receiving antipsychotics, antiepileptic drugs, or opioid analgesics, had obstructive sleep apnea syndrome, had a Body Mass Index > 30, or had undergone laparotomy instead of laparoscopic surgery were excluded. For the 333 Japanese patients who underwent laparoscopic surgery for benign gynecological disease, the recruitment of subjects, surgical protocol, and subsequent postoperative pain management were fundamentally the same as in a previous report [55]. Because the genotypes of one patient were unknown, a total of 332 cases were analyzed. Detailed demographic and clinical data of the subjects are provided in Supplementary Dataset S1.

### 4.3. Pain Assessment

The presence or absence of dysesthesia in the TD sample was assessed based on the definition of the International Association for the Study of Pain. Subjective symptoms were assessed by interview 4 weeks post-surgery. The patients were asked to select words from the McGill Pain Questionnaire [56] to describe their pain (i.e., temporal, brightness, thermal, dullness, traction pressure, constrictive pressure, etc.) [8].

NRS pain scores in the JUH sample were assessed every 3 h (unless patients were asleep) until 12 h postoperatively and then every 6 h until 24 h postoperatively. The NRS pain scores were rated on an 11-point scale, from 0 to 10. The mean NRS pain score in individuals was calculated from these NRS pain scores [55].

### 4.4. Genotyping

The present study examined the rs1051931 SNP of the *PLA2G7* gene. Genomic DNA was extracted from whole-blood samples using standard procedures. The extracted DNA was dissolved in TE buffer (10 mM Tris-HCl and 1 mM ethylenediaminetetraacetic acid, pH 8.0), as described previously [57,58]. Briefly, whole-genome genotyping was performed using Infinium Assay II and the iScan System (Illumina, San Diego, CA, USA) according to the manufacturer’s instructions. Five kinds of BeadChips (HumanHap300, total markers: 317,503; HumanHap300-Duo, total markers: 318,237; Human610-Quad v1, total markers: 620,901; Human1M v1.0, total markers: 1,072,820; Human 1M-Duo v3, total markers: 1,199,187) were used for genotyping 40, 67, 6, 120, and 128 samples, respectively, in the TD group. The Infinium Asian Screening Array-24 v. 1.0 BeadChip (total markers: 659,184) was used for genotyping 333 patients’ samples in the JUH group that remained after the filtration process based on the exclusion criteria of the clinical data. Some BeadChips included a few probes that were specific to copy number variation markers, but most were for SNP markers on the human autosome or sex chromosome. Approximately 300,000 SNP markers were commonly included in all five kinds of BeadChips that were used for genotyping in the TD group. Genome Studio with the Genotyping v. 3.3.7 or v. 2.0.4 module (Illumina) was used to examine data for samples that had their entire genomes genotyped to assess quality of the findings, and quality control was performed as described in our previous report [57]. Because the genotype data for the *PLA2G7* rs1051931 SNP were included in the aforementioned BeadChips for whole-genome genotyping, the data were extracted from the results of whole-genome genotyping and used for the association study.

### 4.5. Statistical Analysis

The statistical analysis was performed using SPSS v. 25 software (IBM Japan, Tokyo, Japan) and Prism 7.00 software (GraphPad, San Diego, CA, USA). We investigated associations between the rs1051931 SNP of the *PLA2G7* gene and dysesthesia in TD samples and NRS pain scores in JUH samples.

Genotype and base description were based on gene orientation. The Kruskal–Wallis test was used to compare continuous variables among patients with the T/T, T/C, and C/C genotypes of the *PLA2G7* rs1051931 SNP. The Mann–Whitney *U* test was used to compare continuous variables between patients with different genotypes or dichotomized genotypes. Categorical data were compared among these patients using the *χ*^2^ test. The Jonckheere–Terpstra trend test and Cochran–Armitage trend test were performed to investigate linear trends. In all statistical tests, the criterion for significance was *p* < 0.05.

### 4.6. Public Database Search

We extracted information on eQTLs of the SNP using the GTEx Portal to examine effects of the SNP on gene expression levels in humans [40]. We extracted information on *PLA2G7* mRNA expression in human tissues and cells using the Human Protein Atlas [44]. We also extracted DNA sequence features plus additional chromatin accessibility (DNase I hypersensitive site [DHS]) and DNase-Seq signals from the genes’ genomic regions using the ZENBU genome browser (accessed 5 February 2025) to investigate transcriptional regulation around SNP regions in the genes [47]. The data source for the DHS was the NIH Roadmap Epigenomics Mapping Consortium for 111 samples, showing promoter, enhancer, and dyadic regions. The data source for the DNase-Seq signals was the NIH Roadmap Epigenomics Mapping Consortium for 127 samples, showing open chromatin regions only with a *p* value signal ≥ 2. Data on promoters and enhancers were derived from DNase I-accessible regulatory regions that were defined by the NIH Roadmap Epigenomics Mapping Consortium. Open chromatin regions correspond to gene expression control regions, such as promoters and enhancers.

## 5. Conclusions

The present study found that the group with the CC genotype of the *PLA2G7* rs1051931 SNP had a higher occurrence of dysesthesia and higher median NRS scores for postoperative pain. In the CC genotype of *PLA2G7* rs1051931, the decrease in PAF-AH activity in plasma may lead to an increase in the amount of PAF in plasma, leading to dysesthesia and postoperative pain. These results provide new insights into individual differences in postoperative pain, including neuropathic pain, based on genetic factors in humans.

## Figures and Tables

**Table 1:** Genotype distribution of *PLA2G7* rs1051931.

Genotype	Group	
	TD	JUH
TT	1 (0.3%)	2 (0.6%)
TC	53 (17.5%)	62 (18.7%)
CC	249 (82.2%)	268 (80.7%)

TD—patients who underwent SSRO at Tokyo Dental College. JUH—patients who underwent laparoscopic surgery at Juntendo University Hospital. The data are expressed as the number (%) of subjects.

**Table 2:** Genotype distribution of *PLA2G7* rs1051931 in patients with and without dysesthesia (TD group).

Genotype Group	Statistical Method	*χ*^2^ Values	*p* Values	With Dysesthesia	Without Dysesthesia
TT/TC/CC	*χ*^2^ test	4.433	0.1090	0/20/129 (0.0/13.4/86.6)	1/33/120 (0.7/21.4/77.9)
	Cochran-Armitage trend test	-	0.0490 *		
TT + TC/CC	*χ*^2^ test	3.873	0.0491 *	20/129 (13.4/86.6)	34/120 (22.1/77.9)
TT/TC + CC	*χ*^2^ test	0.971	0.3245	0/149 (0.0/100.0)	1/153 (0.6/99.4)

* *p* < 0.05. The data are expressed as the number (%) of subjects.

**Table 3:** Association analysis of *PLA2G7* rs1051931 with clinical data (JUH group).

Genotype Group	Numerical Rating Scale Pain Score (Median [Interquartile Range])	Statistical Method	*p* Values
TT/TC/CC	1.3 [0.3]/1.5 [1.8]/1.7 [1.4]	Kruskal–Wallis test	0.0755
		Jonckheere–Terpstra test	0.0243 *
TT + TC/CC	1.5 [1.7]/1.7 [1.4]	Mann–Whitney *U* test	0.0249 *
TT/TC + CC	1.3 [0.3]/1.7 [1.5]	Mann–Whitney *U* test	0.4706

* *p* < 0.05.

## Data Availability

The raw data in this study are shown in Supplementary Dataset S1.

## Supplementary Materials

The following supporting information can be downloaded at https://www.mdpi.com/article/10.3390/ijms26093931/s1.
