# Supplementary material for: rs1051931 Nonsynonymous Polymorphism of Platelet-Activating Factor Acetylhydrolase Gene PLA2G7 Is Associated with Dysesthesia and Pain Severity After Surgery
Source: Int J Mol Sci. 2025 Apr 22;26(9):3931. doi: 10.3390/ijms26093931 (PMC12071393; doi:10.3390/ijms26093931)
Supplement: Supplementary file 1 [file ijms-26-03931-s001.zip › Hayashi et al Supplemental Figure S1.pdf]

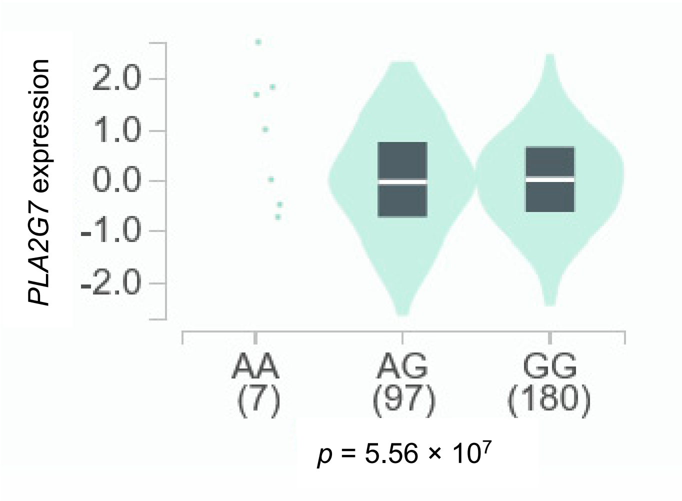

Figure S1. *PLA2G7* rs1051931 is an eQTL for *PLA2G7*.

PLA2G7 expression levels are shown for each genotype of the *PLA2G7* rs1051931 SNP in the nucleus accumbens (basal ganglia; eQTL: $p = 5.56 \times 10^{-7}$; GTEx portal). The normalized effect size was -0.25, indicating that *PLA2G7* mRNA expression in AA, AG, and GG genotypes decreased in order.
